# Supplementary material for: Waves of chromatin modifications in mouse dendritic cells in response to LPS stimulation
Source: Genome Biol. 2018 Sep 19;19:138. doi: 10.1186/s13059-018-1524-z (PMC6146659; doi:10.1186/s13059-018-1524-z)
Supplement: Supplementary file 1 — Contains supplementary results, supplementary methods, supplementary tables, and supplementary figures. (PDF 5904 kb) [file 13059_2018_1524_MOESM1_ESM.pdf]

Supplementary material for “Waves of chromatin modifications in mouse dendritic cells in response to LPS stimulation”

Authors: Alexis Vandenbon[1,2$]*, Yutaro Kumagai[3,4$], Mengjie Lin[5], Yutaka Suzuki[5], Kenta Nakai[6]*

[1] Laboratory of Infection and Prevention, Institute for Frontier Life and Medical Sciences, Kyoto University, Kyoto 606-8507, Japan

[2] Institute for Liberal Arts and Sciences, Kyoto University, Kyoto 606-8507, Japan

[3] Quantitative Immunology Research Unit, Immunology Frontier Research Center (IFReC), Osaka University, Suita, 565-0871, Japan

[4] Biotechnology Research Institute for Drug Discovery, Department of Life Science and Biotechnology, National Institute of Advanced Industrial Science and Technology, Tsukuba, Ibaraki 305-8565, Japan (current affiliation)

[5] Department of Computational Biology and Medical Sciences, Graduate School of

Frontier Sciences, The University of Tokyo, Kashiwa 277-8561, Japan

[6] Laboratory of Functional Analysis in silico, The Institute of Medical Science, The University of Tokyo, Minato-ku, Tokyo, 108-8639, Japan

$ Equal contribution

* To whom correspondence should be addressed. Email: alexisvdb@infront.kyoto-u.ac.jp, knakai@ims.u-tokyo.ac.jp

Table of Contents

Supplementary Results 2

Analysis of histone modification changes in LPS-treated macrophages 2

a) Overlap in loci with changes in LPS-treated macrophages 2

b) Timing of changes in LPS-treated macrophages 2

A Subset of STAT1/2 Target Genes lack Induction of H3K9K14ac and H3K4me3 in *Trif*$^{-/-}$, *Irf3*$^{-/-}$, and *Ifnar1*$^{-/-}$ cells 2

Supplementary Methods 3

Deciding the main TSS of promoter regions 3

Primer sequences 4

Analysis of public ChIP-seq data of macrophages 5

Supplementary References 6

Supplementary Figures 7

Supplementary Results

Analysis of histone modification changes in LPS-treated macrophages

To support our findings on LPS-induced dynamics and their timing in DCs, we analyzed a public ChIP-seq dataset for H3K4me1, H3K4me3, H3K27ac, and Pol2 binding in a similar cell type (macrophages) spanning a similar time frame (0, 4, 24h) after LPS stimulation [1].

a) Overlap in loci with changes in LPS-treated macrophages

Of the total of 520 promoters with LPS-induced increases in Pol2 binding in our data, 293 (56.3%) had increases in Pol2 binding in the LPS-treated macrophages data (Fig. S3A). Likewise, a high overlap was seen for promoters with increases in H3K27ac (27.0%) and H3K4me3 (26.7%). Similarly, at enhancers (Fig. S3B), there was a high overlap in the regions with increases in Pol2 binding (32.5%), and H3K27ac (34.5%). Increases in H3K4me3 at enhancers were rare in our dataset, but nevertheless overlapped with those in the LPS-treated macrophages (23.1%).

In striking contrast, loci with increases in H3K4me1 in our data overlapped little with those in the macrophage data. Of the total 774 promoters with increases in H3K4me1 levels in our data, only 6 (0.8%) had an increase in the Ostuni *et al.* data. Overlap of increases in H3K4me1 at enhancers too was markedly lower than that of H3K27ac and H3K4me3, although still significantly higher than expected (14.3%, $p < 1e\text{-}4$). Possibly this reflects differences between DC- and macrophage-specific enhancers and the molecular processes that define these cell types.

b) Timing of changes in LPS-treated macrophages

Focusing on loci overlapping with promoters and enhancers as defined in our dataset (see section "Analysis of public ChIP-seq data of macrophages"), we observed similar tendencies in timing of increases (Fig. S13A): increases in H3K27ac occurred preferentially during the first 4 hours after stimulation (893 out of 1,073 promoters, 83.2%). The majority of increases in H3K4me3 too occurred during the first 4 hours (355 out of 559 promoters, 63.5%). In contrast, accumulation of H3K3me1 (which was rare) and increases in Pol2 binding were spread more evenly over the time course (keeping in consideration the length of the time windows: 0 to 4 hours and 4 to 24 hours). These results too, suggest that increases in H3K27ac and – to a lesser extent – H3K4me3 at promoters occur early after stimulation.

At enhancers too, increases in H3K27ac were early (77.0%, Fig. S13B), while accumulation of H3K4me1 was spread more evenly (18.7% early). In striking contrast with promoters, however, binding by Pol2 increased earlier (72.6% for enhancers versus 39.6% for promoters). Increases in H3K4me3 were rare at enhancers, but - interestingly - had a tendency to happen between the 4 and 24 hour time points.

A Subset of STAT1/2 Target Genes lack Induction of H3K9K14ac and H3K4me3 in *Trif*$^{-/-}$, *Irf3*$^{-/-}$, and *Ifnar1*$^{-/-}$ cells

A set of known TRIF-independent genes (*Tnf*, *Il1b*, *Cxcl1*, and *Nfkbiz*) had no change between WT and knock outs (KOs) in the induction of gene expression, H3K9K14ac and H3K4me3 (Fig. S11A). These genes are not bound by STAT1/2 (except for *Cxcl1* which is bound only by STAT2), and the induction of histone modifications at their promoter regions is likely to be STAT1/2-independent.

A second subset of LPS-induced genes (*Ifit1* and *Rsad2*) has TRIF-dependent expression. These genes become bound by STAT1/2 2 hours after LPS stimulation. Their expression and the induction of H3K9K14ac and H3K4me3 were completely abrogated in *Trif*$^{-/-}$ cells, in *Irf3*$^{-/-}$ cells, and in *Ifnar1*$^{-/-}$ cells (Fig. S11B). These results further support a role of STAT1 and/or STAT2 in the control of chromatin modifications at the promoters of these genes.

A third subset of genes (*Cxcl10*, *Ccl5*, and *Il6*) was partially dependent on TRIF, IRF3, and IFNR in their induction of gene expression and histone modification changes (Fig. S11C). Induction of H3K4me3 was abrogated for *Cxcl10* in all three KOs, although H3K9K14ac was only affected in *Trif*$^{-/-}$cells. Modifications at *Ccl5* and *Il6* too, showed a dependency of TRIF, but not on IFNR. Although these three genes were bound by STAT1 or STAT2 2 hours after LPS stimulation, it is likely that their gene expression and histone modifications are regulated by additional, partly redundant signaling pathways downstream of TLR4 [2]. Indeed, RelA and IRF1 bind to the promoters of these genes, supporting the notion of combinatorial control of gene expression and histone modifications.

Furthermore, stimulation of WT cells using IFN-β induced expression of *Ifit1* and *Rsad2*, and accumulation of H3K9K14ac and H3K4me3 at their promoters (Fig. S22B). In this system, the activation of the IFNR signaling pathway, and of STAT1/2, is independent of TRIF. Accordingly, this accumulation of H3K9K14ac and H3K4me3 was not affected in *Trif*$^{-/-}$ cells, further supporting a role for STAT1/2 in the control of these modifications at these genes. Similar accumulations were observed for *Cxcl10*, *Ccl5*, and *Il6* (Fig. S22C). In contrast, no or only limited accumulation was observed for *Tnf*, *Il1b*, *Cxcl1*, and *Nfkbiz* (Fig. S22A).

Supplementary Methods

Deciding the main TSS of promoter regions

In many TSS-seq based promoter regions, there are several candidate TSSs (bases with aligned TSS-seq reads). We used a log-likelihood model for deciding the representative TSS for each promoter region, inspired by position-specific scoring matrices used for modeling binding specificities of TFs [3]. In brief, we constructed a model capturing properties that are typical for TSSs (as compared to randomly selected loci), and use the model to score candidate TSSs. For each promoter with multiple candidate TSSs, the TSS with the highest score for our model was selected. More details are explained below.

For each biological feature f (here: histone modifications, Pol2 binding, RNA-seq reads, and TSS-seq reads) in our dataset, we averaged aligned read counts (ppm; reads per million reads) over the ten time points. Next, we calculated $ppm_{x,f,i}$, being the number of reads (in ppm) around the main TSS for each promoter x (i.e. the base with the highest number of aligned TSS-seq reads) in bin i, for 20 bins of 100 bps over the region -1 kb to + 1kb around the main TSS.

From the ppm values in all bins, we generated a position probability matrix M_f for each feature f, as follows:

$$M_f[i,j] = \frac{1}{n} \sum_{x \in P} I\left(ppm_{x,f,j} \in bin_i\right) \quad (1)$$

where $M_f[i,j]$ is the entry on row i and column j in M_f, n is the number of promoter regions in the genome-wide set of TSS-seq derived promoters P, and $I(ppm_{x,f,j} \in bin_i)$ is 1 if the value $ppm_{x,f,j}$ is within the range specified by bin_i (otherwise it is 0). To decide the ranges of bin_i, for each feature f, six equally populous bins were decided based on all $ppm_{x,f,j}$ values.

M_f captures the typical signal for feature f around TSSs. We similarly generated a control, M_f^{rand}, based on 10,000 randomly selected genomic regions, using the same bin values as used for the TSS-seq derived promoters. From M_f and M_f^{rand}, a position weight matrix was generated as follows:

$$PWM_f = ln\left({}^{M_f}\!/_{M_f^{rand}}\right) \quad (2)$$

This PWM_f represents a log-likelihood model for feature f in promoters as compared to control sequences (randomly selected regions). Using these models, for each promoter region x, we scored all candidate TSSs t by summing over the relevant bin for each position j (the 20 bins of 100 bps):

$$score_{t,f} = \sum_{j=1}^{20} PWM_f\,[i,j] \quad (3)$$

where i is the index of bin_i range corresponding to the value of $ppm_{x,f,j}$. Thus we obtained a total score over all features f as follows:

$$score_{t,total} = \sum_{f \in features} score_{t,f} \quad (4)$$

where $features$ include all histone modifications, Pol2 binding, RNA-seq reads, and TSS-seq reads.

The final representative TSS $\hat{t}$ of promoter region x was defined as the TSS having the highest total score. In practice, for each promoter we considered at most 10 bases with the highest count of aligned TSS-seq reads as candidate TSSs. Moreover, candidate TSSs with few aligned TSS-seq reads (less than one tenth of the number of TSS-seq reads aligned to the base with the highest number of reads) were not considered. The thus decided representative TSSs were used as the “center” of each promoter for the downstream analysis of increases in histone modifications (see main manuscript).

In addition to deciding the representative TSS for each promoter region, this model was also used for removing low scoring promoters (e.g. regions with only a TSS-seq signal, lacking Pol2 binding signals, lacking histone modifications typical of promoters, etc). For this, we removed promoters with total scores < 0.

Finally, for the assignment of main promoters to genes, if a gene had multiple candidate promoter regions, we assigned to it the one with the highest total score for our model.

Primer sequences

Gene (forward/reverse)	Primer sequence
Actb (fw)	GGGAATGGGTCAGAAGGACT
Actb (rv)	CTTCTCCATGTCGTCCCAGT
Il6 (fw)	AGTTGCCTTCTTGGGACTGA
Il6 (rv)	ACAGGTCTGTTGGGAGTGGT
Tnf (fw)	CCCCAAAGGGATGAGAAGTT

Tnf (rv)	CACTTGGTGGTTTGCTACGA
Il1b (fw)	TGAAGCAGCTATGGCAACTG
Il1b (rv)	GGGTCCGTCAACTTCAAAGA
Cxcl1 (fw)	ACTCCAACACAGCACCATGA
Cxcl1 (rv)	ATGGTCTGCAGGCACTGAC
Cxcl10 (fw)	AAGTGCTGCCGTCATTTTCT
Cxcl10 (rv)	CCTATGGCCCTCATTCTCAC
Ifit1 (fw)	CAAGGCAGGTTTCTGAGGAG
Ifit1 (rv)	CATTCTCTCCCATGGTTGCT
Rsad2 (fw)	ACAGCCAAGACATCCTTCGT
Rsad2 (rv)	TCTTCTCCAAACCAGCCTGT
Nfkbiz (fw)	AGAAAGGGACCCGATCCTC
Nfkbiz (rv)	CGGTGATGTCACGAAGTGAG
Ccl5 (fw)	CGAGGGAGAGGTAGGCAAAG
Ccl5 (rv)	TCACCATCATCCTACCTGCA

Table S1: Table of primer sequences used for RT-qPCR.

Gene (forward/reverse)	Primer sequence
Tnf (fw)	GTGCCTATGTCTCAGCCTCT
Tnf (rv)	CCAGACACTCACCTCATCCC
Il1b (fw)	CACTGATGGACTTTGGGCTT
Il1b (rv)	TGTCAGCGGCTATACAGACA
Cxcl1 (fw)	CTATCGCCAATGAGCTGCG
Cxcl1 (rv)	GACTTCGGTTTGGGTGCAG
Nfkbiz (fw)	CGGCGAGCTCTAGAGAAAGA
Nfkbiz (rv)	CCCCAAGTACGTGAGAGCAT
Ifit1 (fw)	CAAGGCAGGTTTCTGAGGAG
Ifit1 (rv)	CCCTCAGAGTGGAGAACAGG
Rsad2 (fw)	AGCAGCCGAGCAGCTAGAG
Rsad2 (rv)	ATAAGCCCTTACAGGCAGCA
Cxcl10 (fw)	CACATGACCATTTCATGTCAGTT
Cxcl10 (rv)	AAAACCGTCCAATACCTTTTGT
Ccl5 (fw)	ACCTGCCTCACCATGTAAGT
Ccl5 (rv)	AGAAGGGGAGGTCTGGGTAT
Il6 (fw)	GAGGAGTGTGAGGCAGAGAG
Il6 (rv)	CTGCGTGGAGAAAAGGGAAA

Table S2: Table of primer sequences used for ChIP-qPCR.

Analysis of public ChIP-seq data of macrophages

H3K4me1, H3K4me3, H3K27ac and Pol2 ChIP-seq data for macrophages treated with LPS (0, 4, and 24 hours) were downloaded from the GEO database (accession number GSE38379). As a control sample we used the same input sample as was used in the original study (accession number GSM499415) [4]. Reads were mapped in the same way as used for our in-house ChIP-seq data. Peaks were predicted in each ChIP sample using the input sample as control, and also in each stimulated sample using the

corresponding untreated samples as control, under the same parameter settings as used in [1]. We set the threshold for significant peaks at p = $1e^{-10}$.

LPS-induced peaks were defined as the regions with a peak in any of the two treated samples compared to the untreated samples which also simultaneously had a peak in the treated sample as compared to the input sample. Early induced peaks were defined as the significant peaks in the 4h sample. Peaks with late induction were defined as induced peaks of the 24h samples that were not overlapping any early induced peak. Intersecting and merging of genomic regions was performed using BEDTools (version 2.17.0) [5]. The overlap between our data and the Ostuni *et al.* data was evaluated using 10,000 randomizations using the BEDTools "shuffle" function. Reported p values (Tables S1 and S2) reflect the fraction of randomizations with higher overlap than the observed overlap.

Supplementary References

1. Ostuni R, Piccolo V, Barozzi I, Polletti S, Termanini A, Bonifacio S, et al. Latent enhancers activated by stimulation in differentiated cells. Cell. 2013;152:157–71.

2. Hirotani T, Yamamoto M, Kumagai Y, Uematsu S, Kawase I, Takeuchi O, et al. Regulation of lipopolysaccharide-inducible genes by MyD88 and Toll/IL-1 domain containing adaptor inducing IFN-beta. Biochem. Biophys. Res. Commun. 2005;328:383–92.

3. Stormo GD. DNA binding sites: representation and discovery. Bioinformatics. 2000;16:16–23.

4. de Santa F, Barozzi I, Mietton F, Ghisletti S, Polletti S, Tusi BK, et al. A large fraction of extragenic RNA Pol II transcription sites overlap enhancers. PLoS Biol. 2010;8.

5. Quinlan AR, Hall IM. BEDTools: A flexible suite of utilities for comparing genomic features. Bioinformatics. 2010;26:841–2.

6. Garber M, Yosef N, Goren A, Raychowdhury R, Thielke A, Guttman M, et al. A High-Throughput Chromatin Immunoprecipitation Approach Reveals Principles of Dynamic Gene Regulation in Mammals. Mol. Cell. 2012;47:810–22.

Supplementary Figures

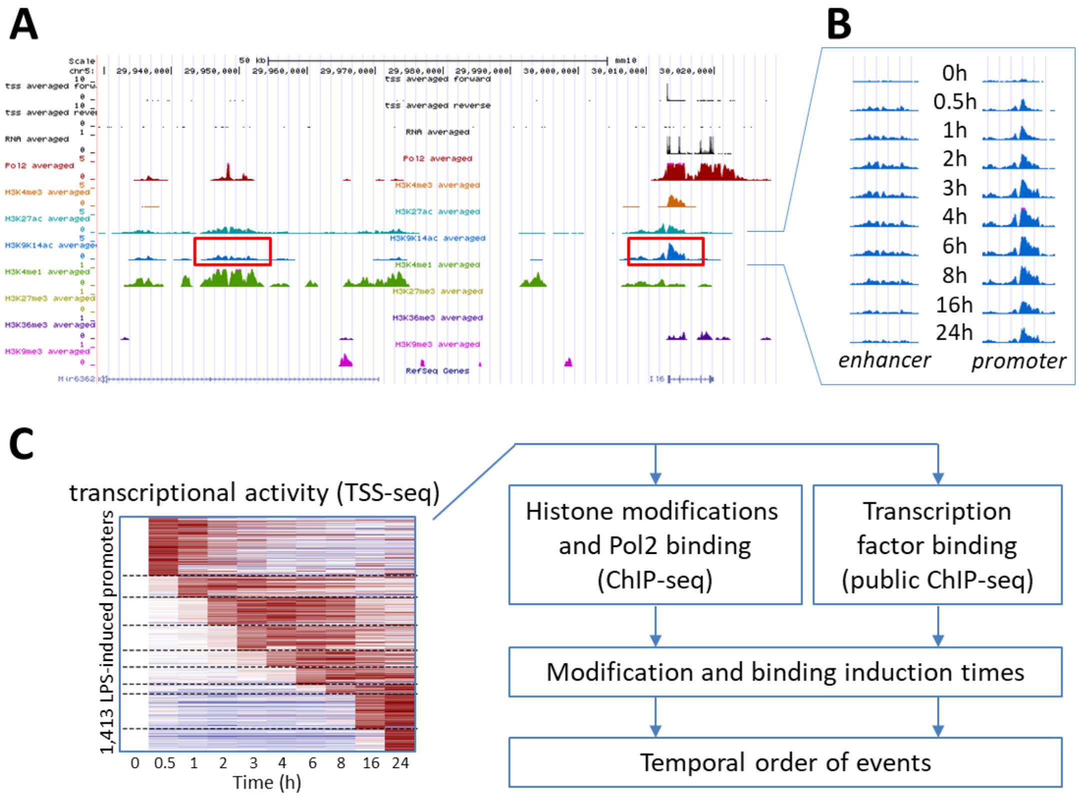

Fig. S1: Overview of data and analysis. (A). As an illustration of our dataset, averaged signals are shown for all features included in this study in the locus including the gene *Il6* and several potential enhancer regions upstream of *Il6*. (B) Detailed view of H3K9K14ac signals over time after LPS stimulation, around the Il6 promoter region and an upstream enhancer region. (C) Focusing on a set of 1,413 LPS-induced promoters, we defined significant induction of histone modifications, Pol2 binding, and TF binding over time. We analyzed the ordering of induction times of histone modifications and binding events, and generated hypotheses regarding underlying molecular mechanisms.

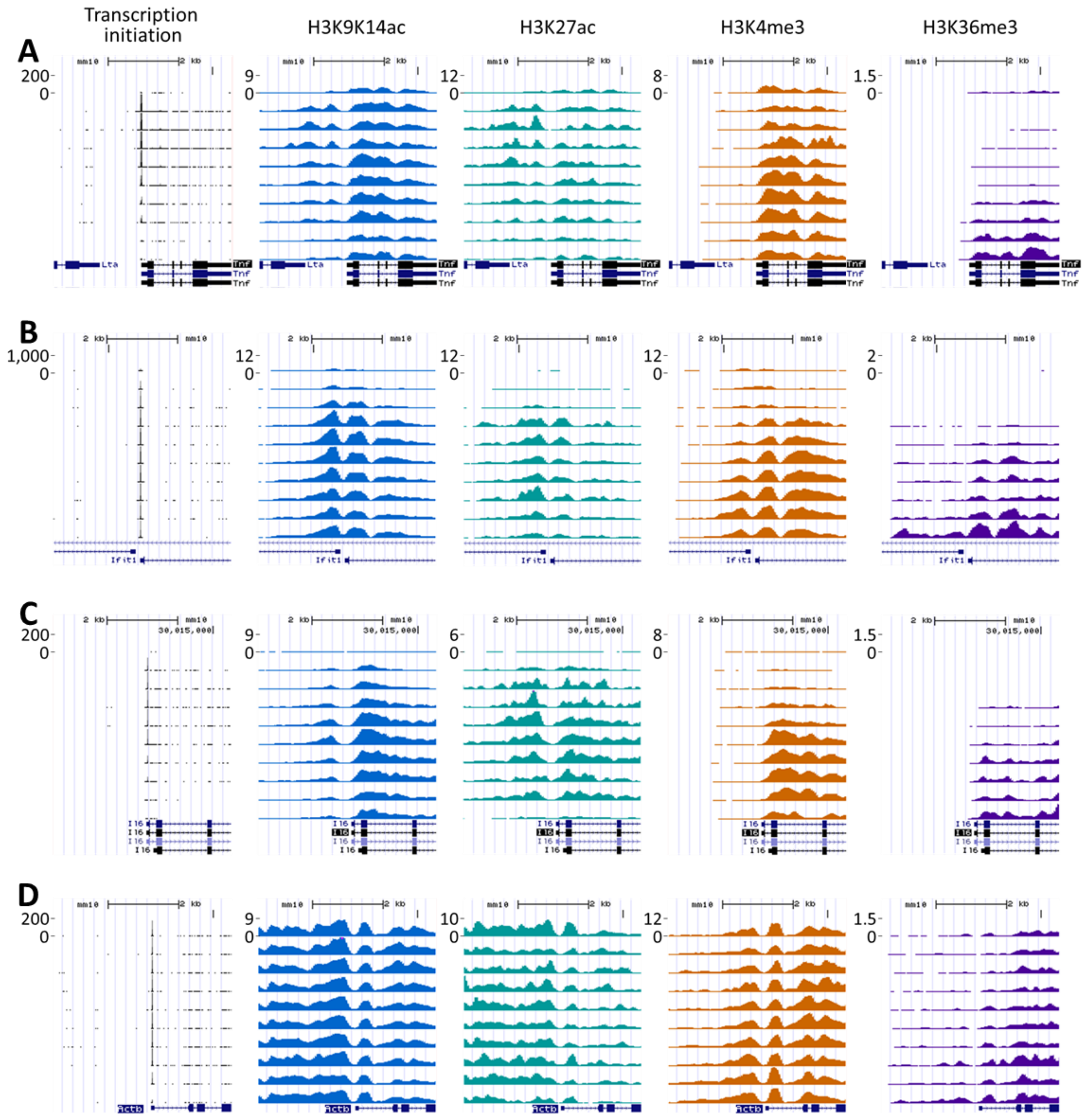

Fig. S2: Snapshots of the data for TSS-seq and ChIP-seq of four histone modifications around the promoters of three LPS-induced genes: **(A)** *Tnf*, **(B)** *Ifit1*, and **(C)** *Il6*, and a gene with no changes in transcription: **(D)** *Actb*. For each feature, all ten time points are shown from 0h (top) to 24h (bottom). For *Tnf*, *Ifit1*, and *Il6,* transcription is induced at 0.5h, but histone modifications accumulate in distinct patterns. *Tnf* has higher basal levels of H3K9K1ac, H3K27ac, and H3K4me3 than *Ifit1* and *Il6*. Modifications remain constant for *Actb*.

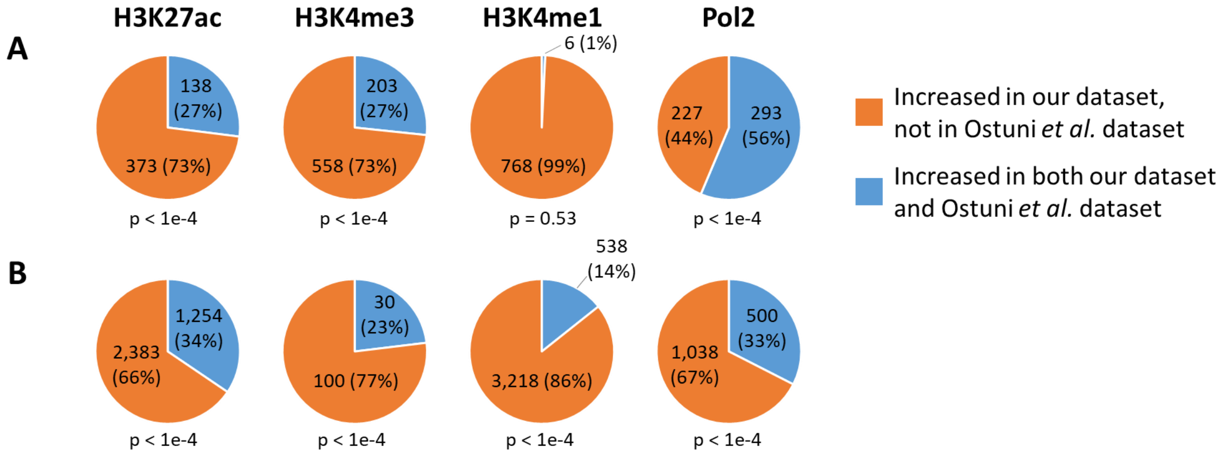

Fig. S3: Overlap in LPS-induced increases at promoters between our data and a dataset obtained from macrophages stimulated with LPS [1]. Pie charts are showing the number (and percentage) of (A) promoters and (B) enhancers with increases in H3K27ac, H3K4me3, H3K4me1, and Pol2 binding. Orange: regions with increases in our dataset only. Blue: regions with increases in both our dataset and the Ostuni *et al.* dataset. P values are based on 10,000 randomizations. The overlap between both datasets was statistically significant for all features, except for H3K4me1 at promoters regions.

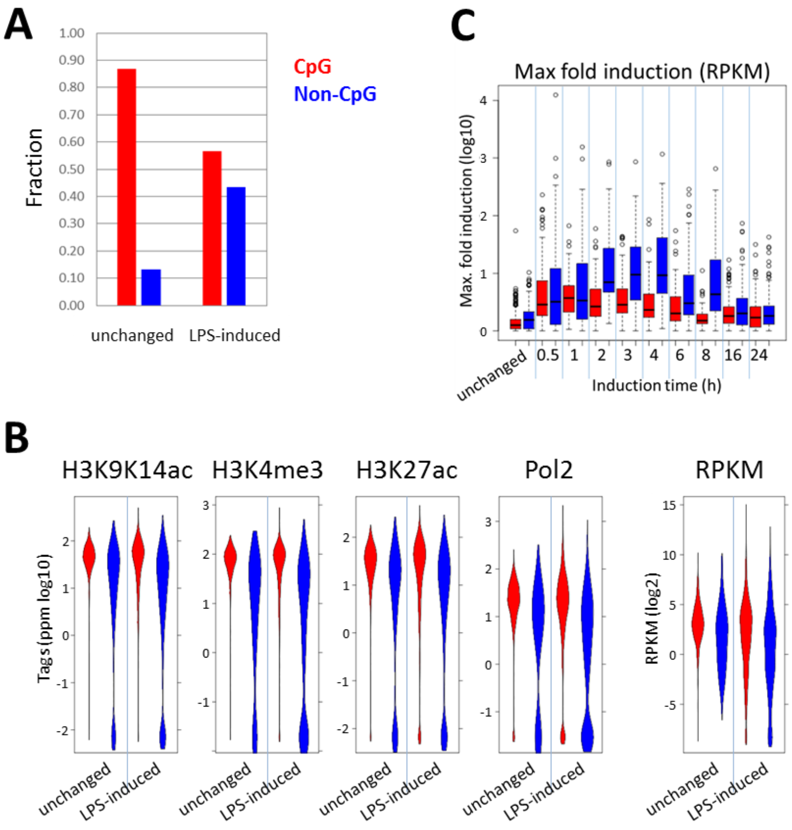

Fig. S4: Basal properties of LPS-induced CpG and non-CpG promoters. (A) Fraction of CpG-associated (red) and non-CpG (blue) promoters per induction time point, and for stable expressed promoters ("unchanged"). (B) Basal (at time 0h) properties of LPS-induced and stably expressed ("unchanged") promoters. A distinction is made between CpG-associated (red) and non-CpG (blue) promoters. Violin plots are shown for basal levels of H3K9K14ac, H3K4me3, H3K27ac, Pol2 binding, and gene expression (RPKMS). (C) Boxplot showing maximum fold-induction of gene expression (RPKM-based) compared to 0h. See Methods section for the definition of induction time.

Fig. S5: (next page) Correlations between basal features at promoters **(A)** and enhancers **(B)**. Features are named on the diagonal. The upper part shows the Pearson correlation coefficient between each pair of features, and the lower part plots pairs of features against each other (ppm in log scale). A small pseudocount was added to avoid problems with 0 values in log scales. At promoters, high correlations were seen especially between Pol2, H3K27ac, H3K9K14ac, and H3K4me3. At enhancers, basal correlations were in general less strong, but relatively high correlations were seen between H3K27ac and H3K9K14ac, Pol2 binding and H3K4me1.

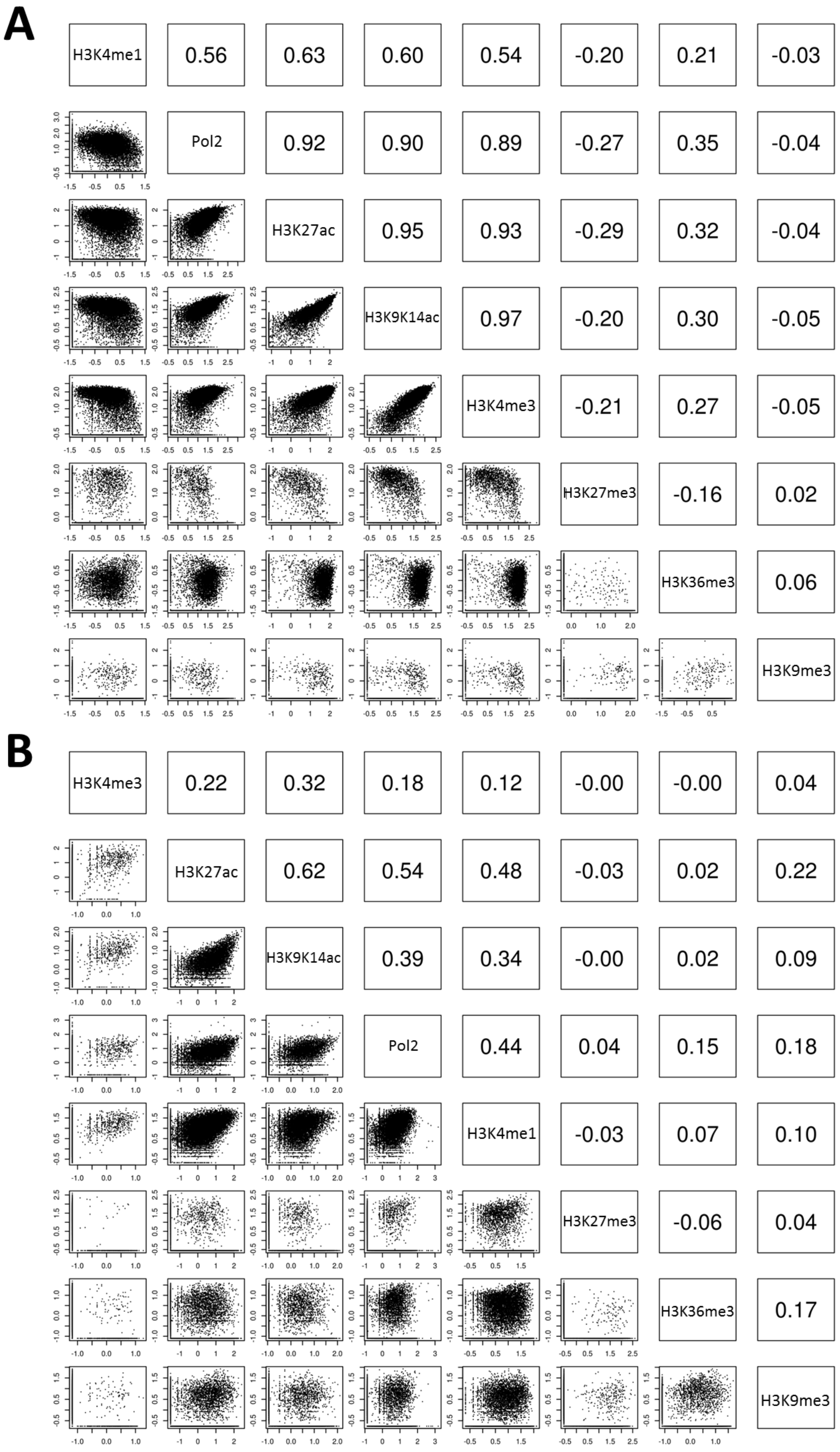
A
H3K4me1
0.56
0.63
0.60
0.54
-0.20
0.21
-0.03
Pol2
0.92
0.90
0.89
-0.27
0.35
-0.04
H3K27ac
0.95
0.93
-0.29
0.32
-0.04
H3K9K14ac
0.97
-0.20
0.30
-0.05
H3K4me3
-0.21
0.27
-0.05
H3K27me3
-0.16
0.02
H3K36me3
0.06
H3K9me3
B
H3K4me3
0.22
0.32
0.18
0.12
-0.00
-0.00
0.04
H3K27ac
0.62
0.54
0.48
-0.03
0.02
0.22
H3K9K14ac
0.39
0.34
-0.00
0.02
0.09
Pol2
0.44
0.04
0.15
0.18
H3K4me1
-0.03
0.07
0.10
H3K27me3
-0.06
0.04
H3K36me3
0.17
H3K9me3

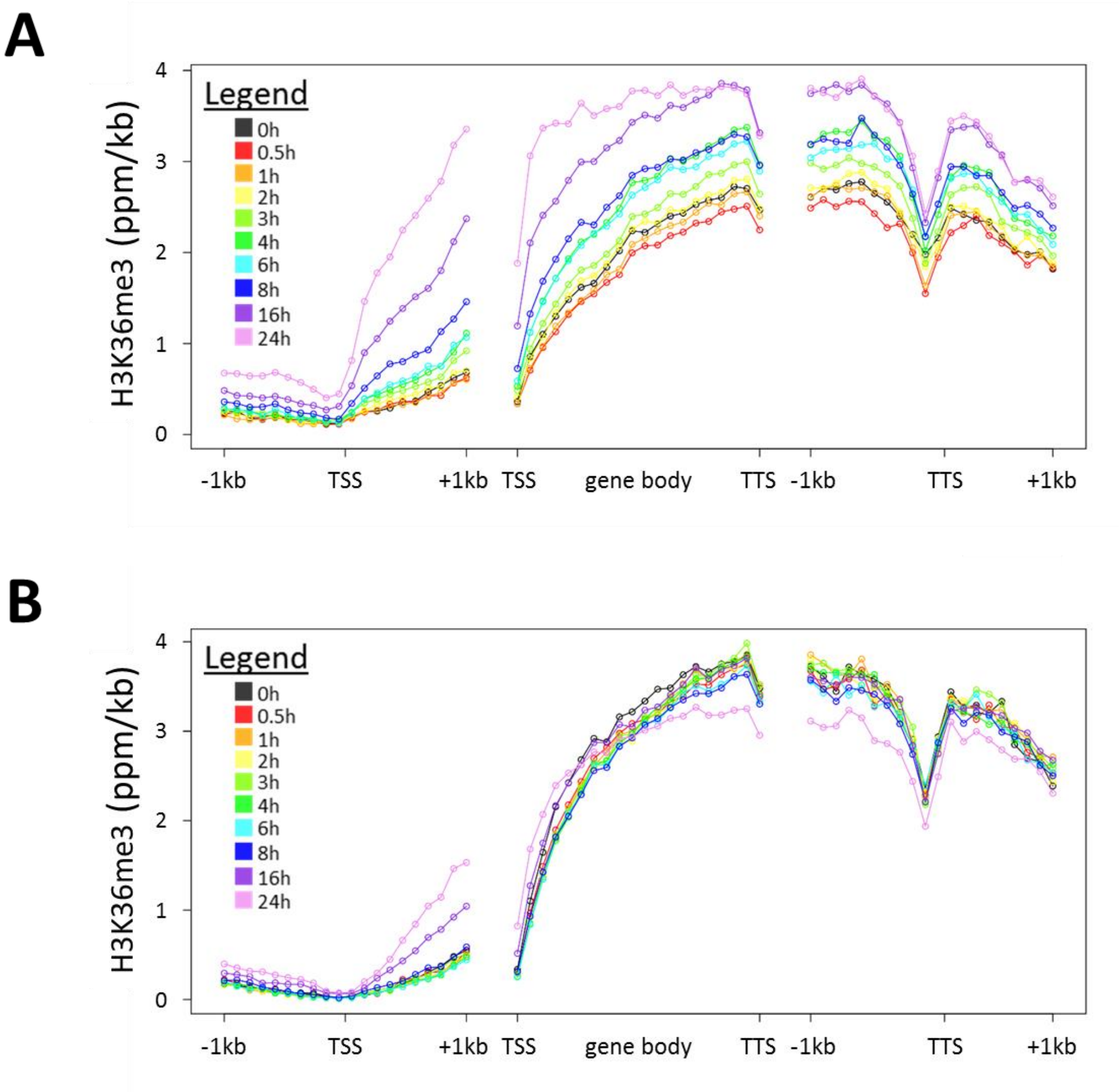

Fig. S6: Changes in H3K36me3 levels at genes' 5' end, gene bodies, and 3' ends. **(A)** Accumulation of H3K36me3 at genes with transcriptional induction after LPS stimulation. The average H3K36me3 signal (ppm/kb) is shown over time for the regions around the transcription start site (TSS) (left, -1kb to +1kb in bins of 100 bps), in the gene body (center, divided into 20 bins of equal size spanning the region from TSS to TTS), and around the transcription termination site (TTS) (right). **(B)** Same as **(A)** for genes with stable expression levels over the time course.

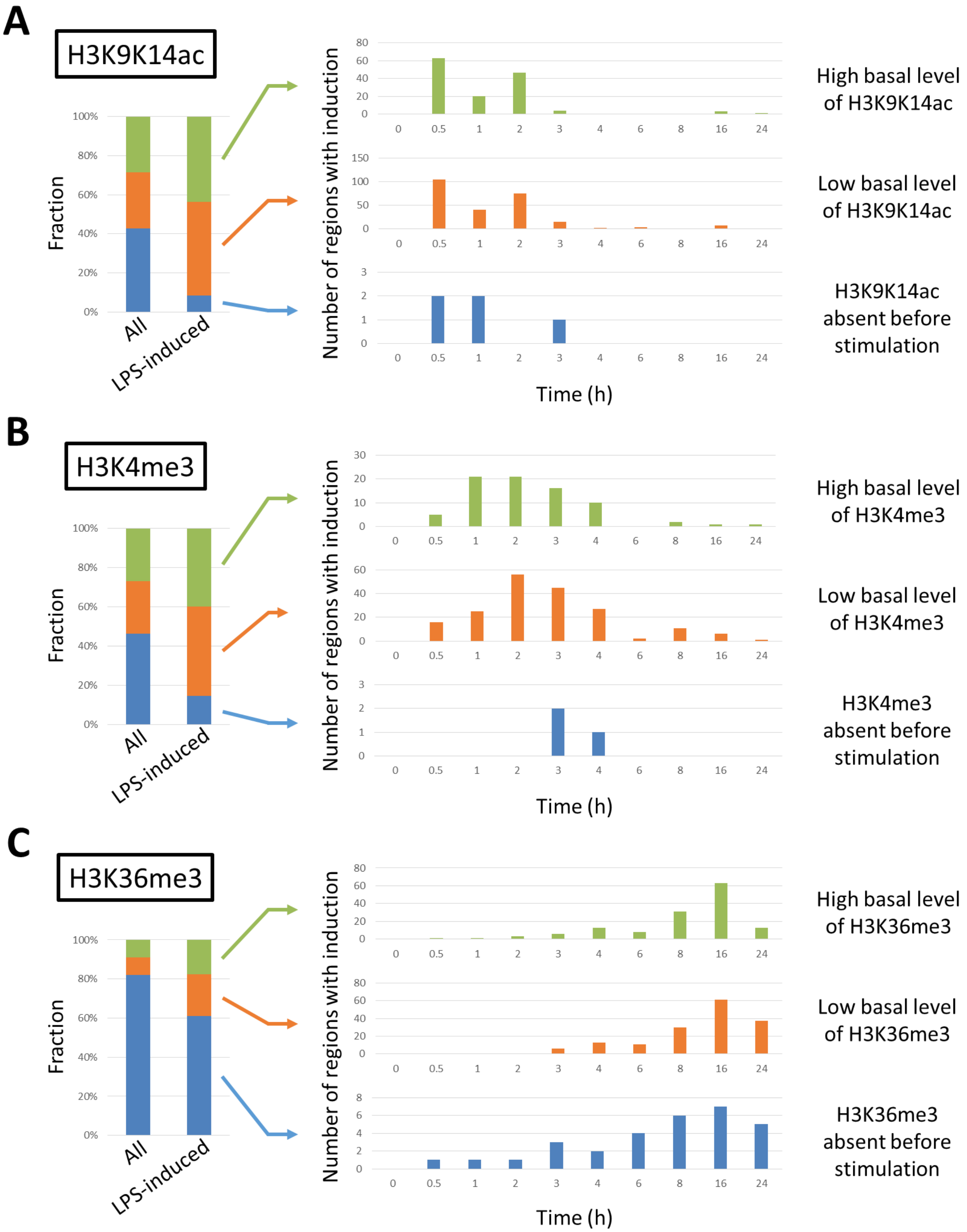

Fig. S7: Induction times of histone modifications in function of their basal levels. **(A)** The genome-wide set of promoters was divided by their pre-stimulation levels of H3K9K14ac into three classes (see Methods). The fraction of promoters in each class is shown at the left, for all promoters, and for LPS-induced promoters. At the right, for each of the three classes, induction times of H3K9K14ac are shown. The same plots are shown for H3K4me3 **(B)**, and H3K36me3 **(C)**.

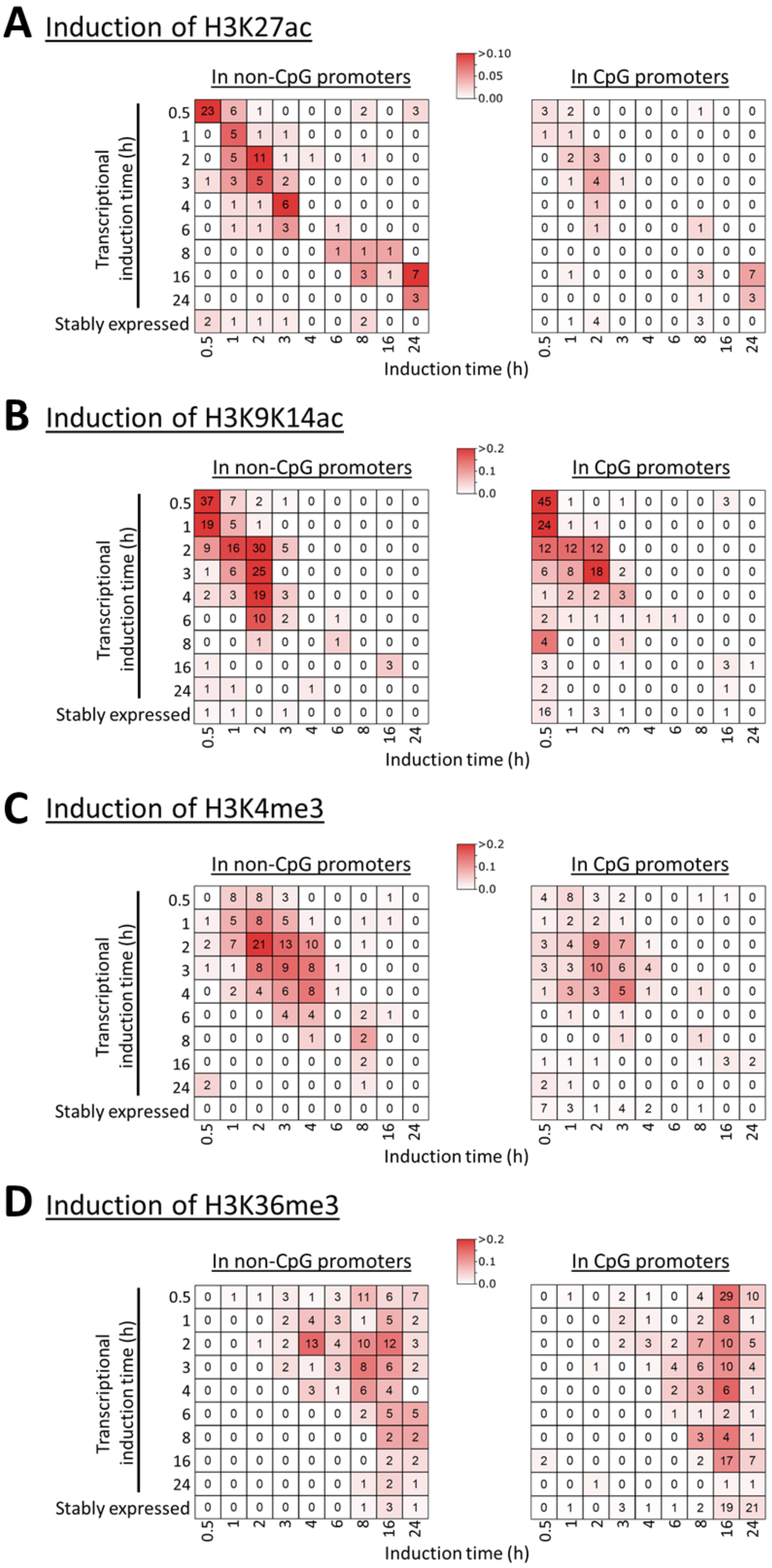
A Induction of H3K27ac
In non-CpG promoters
In CpG promoters
>0.10
0.05
0.00
Transcriptional induction time (h)
Stably expressed
Induction time (h)
B Induction of H3K9K14ac
In non-CpG promoters
In CpG promoters
>0.2
0.1
0.0
Transcriptional induction time (h)
Stably expressed
Induction time (h)
C Induction of H3K4me3
In non-CpG promoters
In CpG promoters
>0.2
0.1
0.0
Transcriptional induction time (h)
Stably expressed
Induction time (h)
D Induction of H3K36me3
In non-CpG promoters
In CpG promoters
>0.2
0.1
0.0
Transcriptional induction time (h)
Stably expressed
Induction time (h)

Fig. S8: (previous page) Induction times of four histone modifications at non-CpG and CpG promoters. Plots are similar to those shown in Fig. 2. (A) H3K27ac, (B) H3K9K14ac, (C) H3K4me3, and (D) H3K36me3. For each pair of plots, the same Y axis scale is used to allow easier comparison of the differences between inductions at non-CpG and CpG promoters.

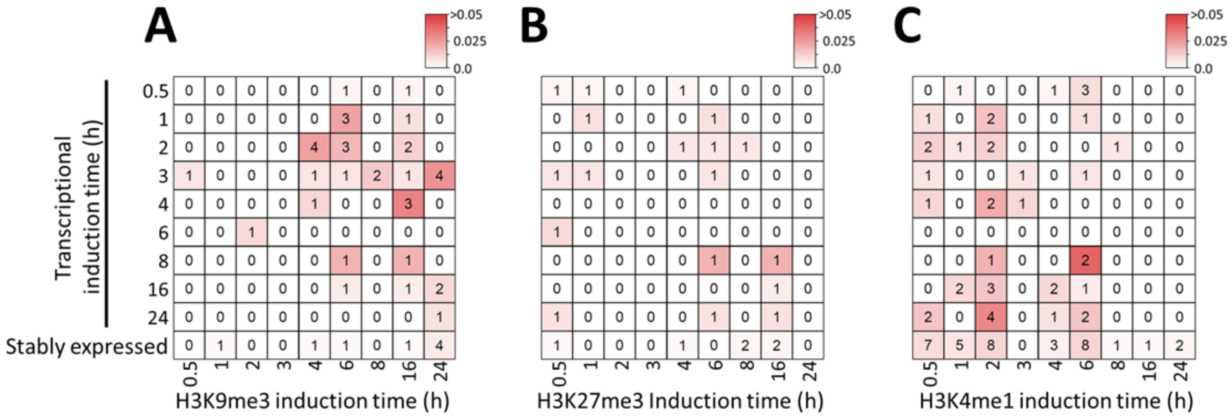

Fig. S9: Induction times of H3K9me3 (A), H3K27me3 (B), and H3K4me1 (C) at promoters in function of induction of transcriptional activation times. Plots are similar to those shown in Fig. 2.

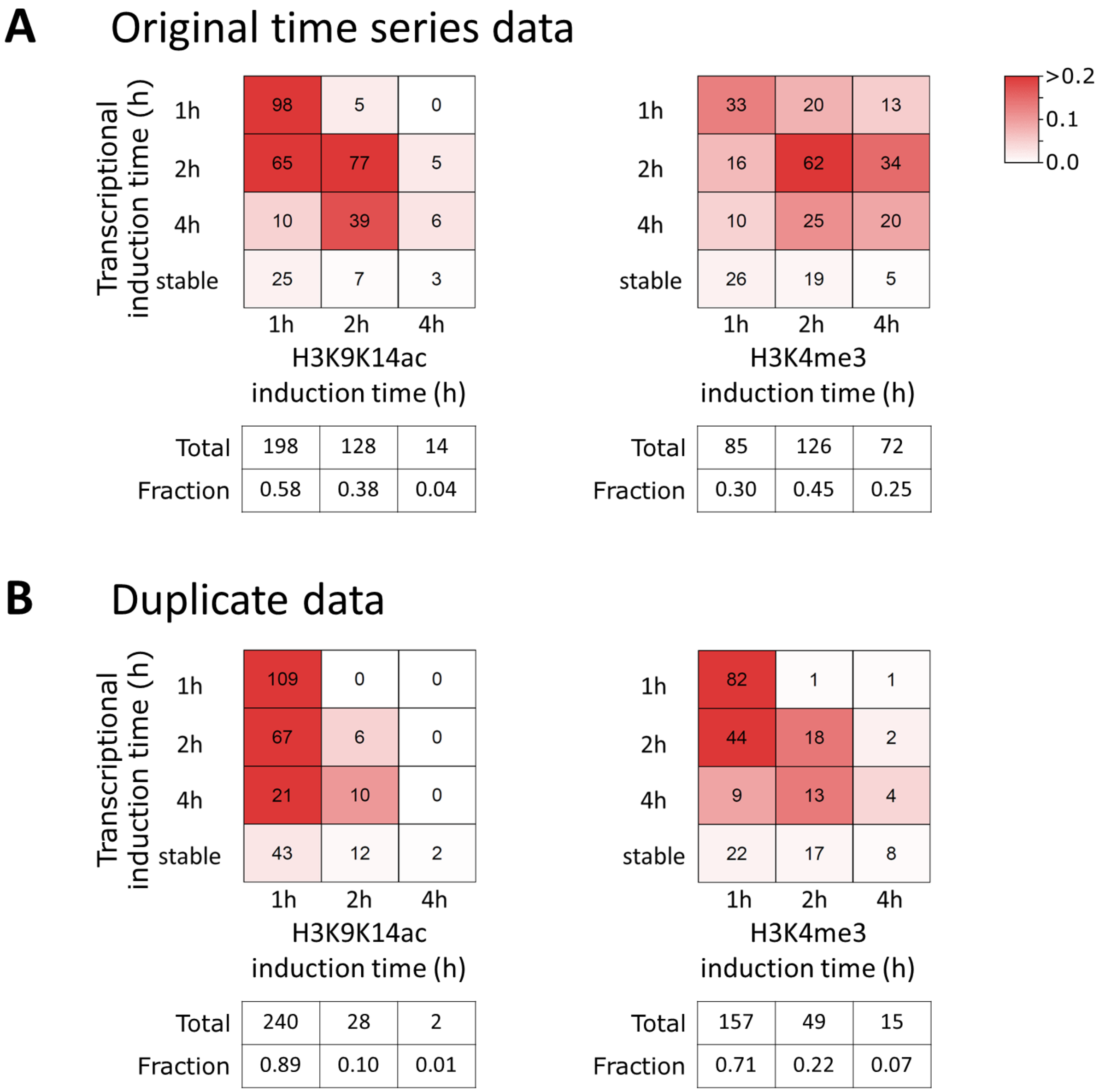

Fig. S10: Induction times of H3K9K14ac and H3K4me3 at LPS-induced promoters (X axis), in function of the transcriptional induction time (Y axis). Data is shown for the original time series dataset (A), and for an independent duplicate time series dataset (B). To facilitate comparison, the original data was re-analyzed using the same time points as the duplicate time series (0, 1, 2, and 4 hours). Below each plot, column sums and fractions of the total are shown. (A) In our original time series, induction of H3K9K14ac was early (between 0.5 and 2h), while induction of H3K4me3 occurred later (between 1 and 4h; see also Fig. 2E,F). (B) In the duplicate time series, too, accumulation of H3K9K14ac is early (mostly at 1h), while H3K4me3 accumulation is seen more frequently at a later time point (1h to 2h). We note that accumulation of both modifications was earlier in the duplicate data than in the original time series, although relative ordering is preserved.

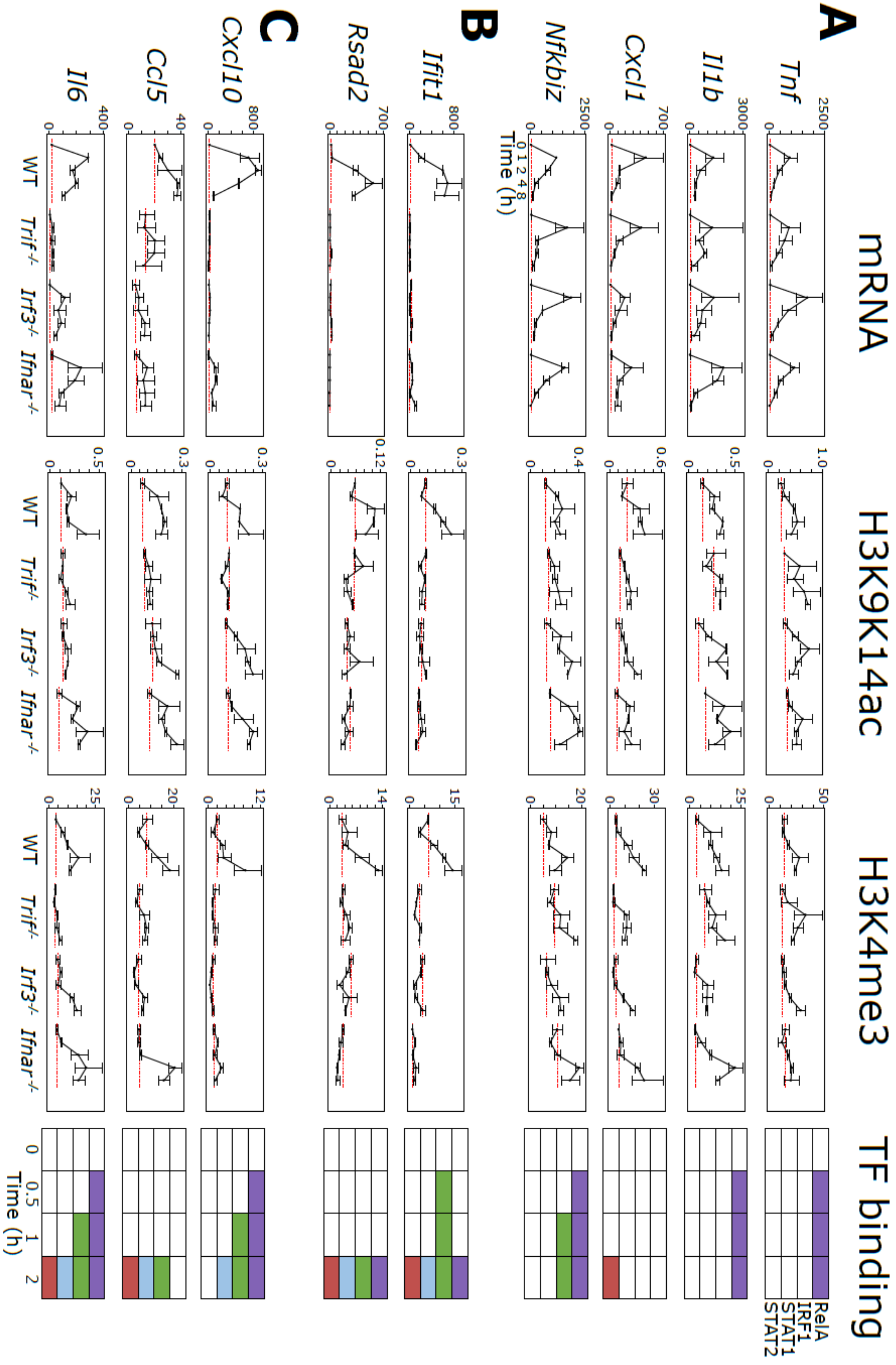
A
B
C
mRNA
H3K9K14ac
H3K4me3
TF binding
Tnf
Il1b
Cxcl1
Nfkbiz
Ifit1
Rsad2
Cxcl10
Ccl5
Il6
WT
Trif-/-
Irf3-/-
Ifnar-/-
Time (h)
RelA
IRF1
STAT1
STAT2

Fig. S11: (previous page) Gene expression (mRNA), H3K9K14ac and H3K4me3 dynamics in WT, *Trif*$^{-/-}$, *Irf3*$^{-/-}$, and *Ifnar*$^{-/-}$ cells following LPS stimulation. We distinguished genes of which expression and histone modifications are independent (A), dependent (B), and partially dependent (C) on TRIF, IRF3, and IFNR. Error bars represent the standard deviation based on duplicate experiments. The red dotted line in each graph represents the mean value at 0h. Y axes represent fold induction (for mRNA) and % input (for H3K9K14ac and H3K4me3). Right column: binding of the promoter of each gene by RelA, IRF1, STAT1 and STAT2 (rows) at time points 0, 0.5, 1, and 2 hours (columns) is indicated, based on ChIP-seq data by Garber *et al*. [6]; white boxes: no binding, colored boxes: binding by RelA (purple), IRF1 (green), STAT1 (blue), and STAT2 (red).

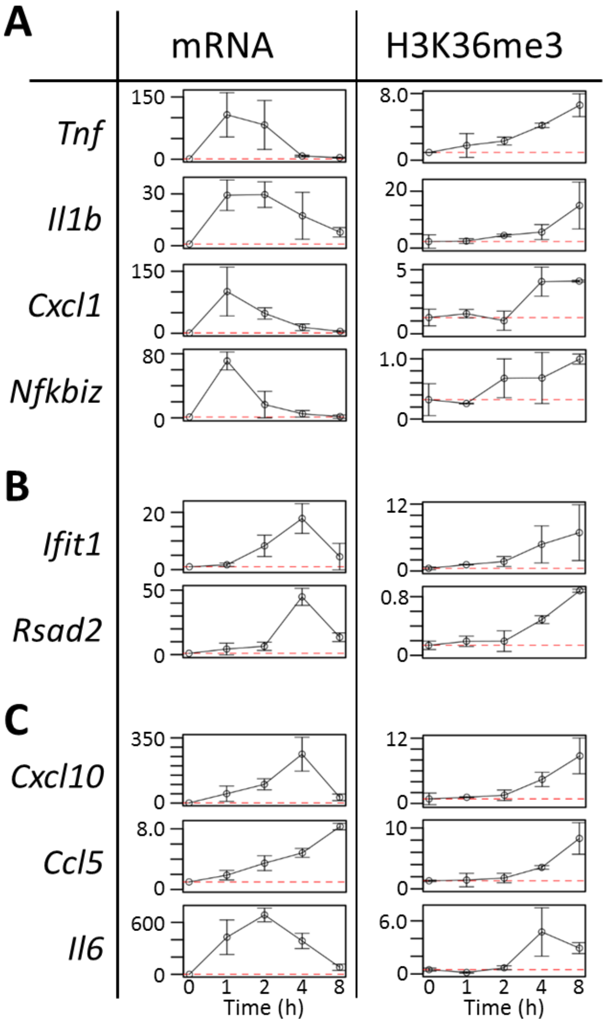

Fig. S12: Gene expression (mRNA) and H3K36me3 dynamics in WT DCs following LPS stimulation. We distinguished genes into the same three groups as described in Fig. S11, according to their dependence on TRIF, IRF3, and IFNR. In all 9 genes, accumulation of H3K36me3 is relatively late compared to induction of gene expression or accumulation of H3K9K14ac and H3K4me3 (see Fig. S11). Error bars represent the standard deviation based on duplicate experiments. The red dotted line in each graph represents the mean value at 0h. Y axes represent fold induction (for mRNA) and % input (for H3K36me3).

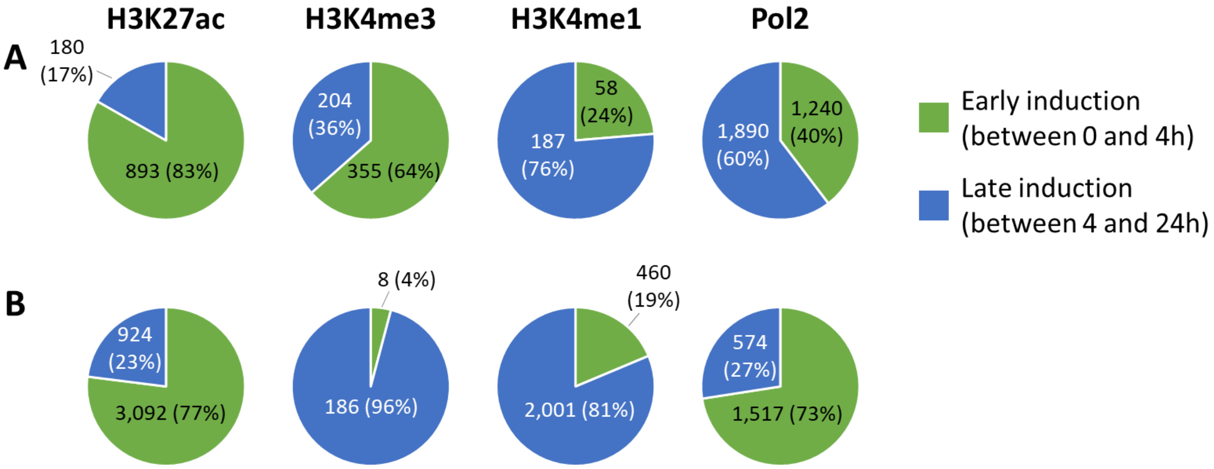

Fig. S13: Timing of increases in H3K27ac, H3K4me3, H3K4me1, and Pol2 binding at (A) promoters and (B) enhancers in LPS-stimulated macrophages [1]. Green: the number (and percentage) of regions with early increases (between time points 0 and 4h). Blue: the number (and percentage) of regions with late increases (between time points 4 and 24h).

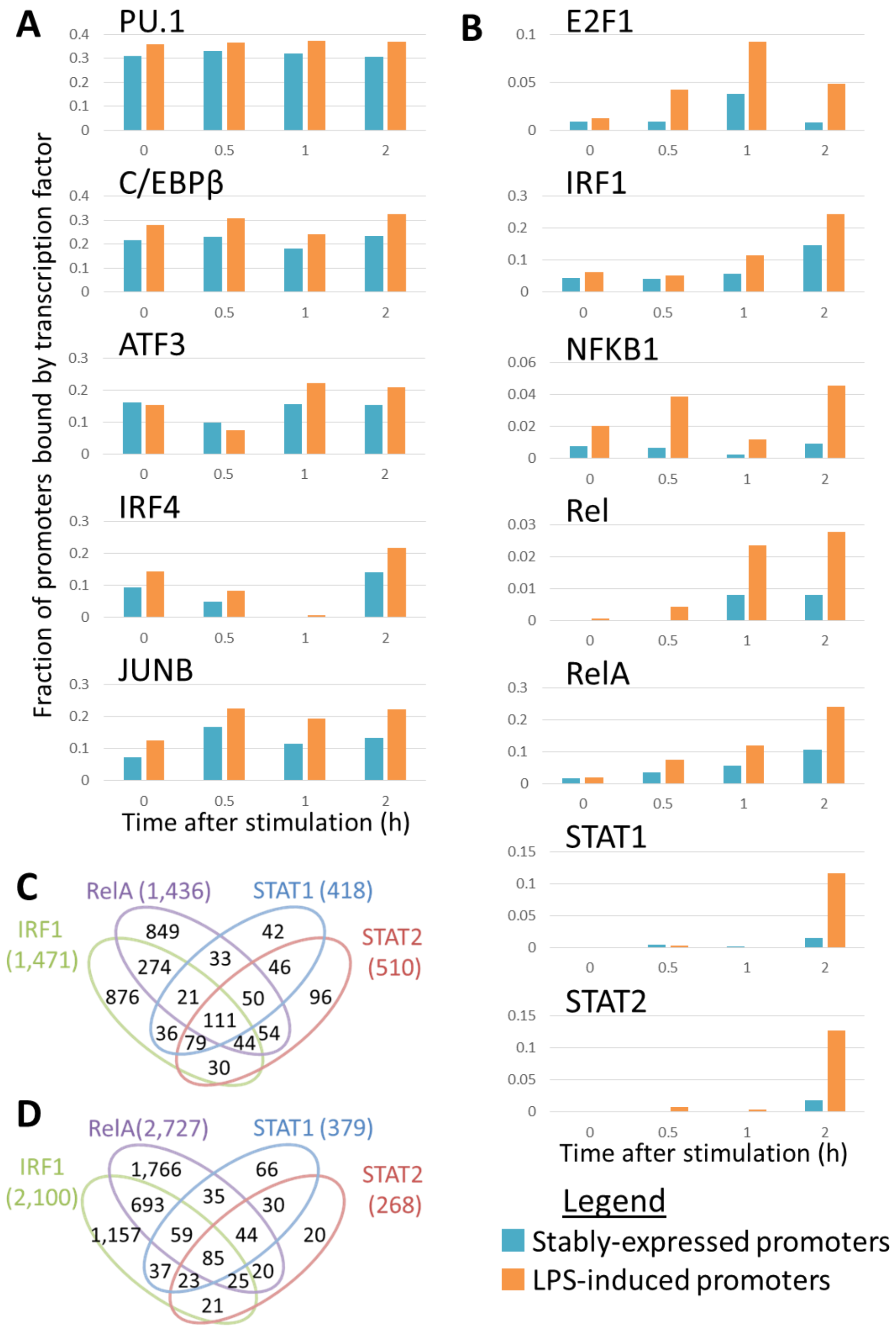

Fig. S14: TF binding in DCs following LPS stimulation. (A-B) For a number of TFs the fraction of promoters bound by each TF is shown in function of time after LPS stimulation. Cyan: stably expressed promoters; orange: LPS-induced promoters. TFs shown in (A) are highly expressed even before LPS stimulation. TFs shown in (B) are LPS-induced. (C-D) Overlap in regions that become bound by IRF1, RelA, STAT1, and STAT2 after LPS stimulation, at promoters (C) and enhancers (D). Numbers in the Venn diagram show the numbers of regions bound by combinations of the four TFs. All combinatorial binding was observed more frequently than expected. Numbers in parentheses show the total number of regions that become bound after stimulation for each TF.

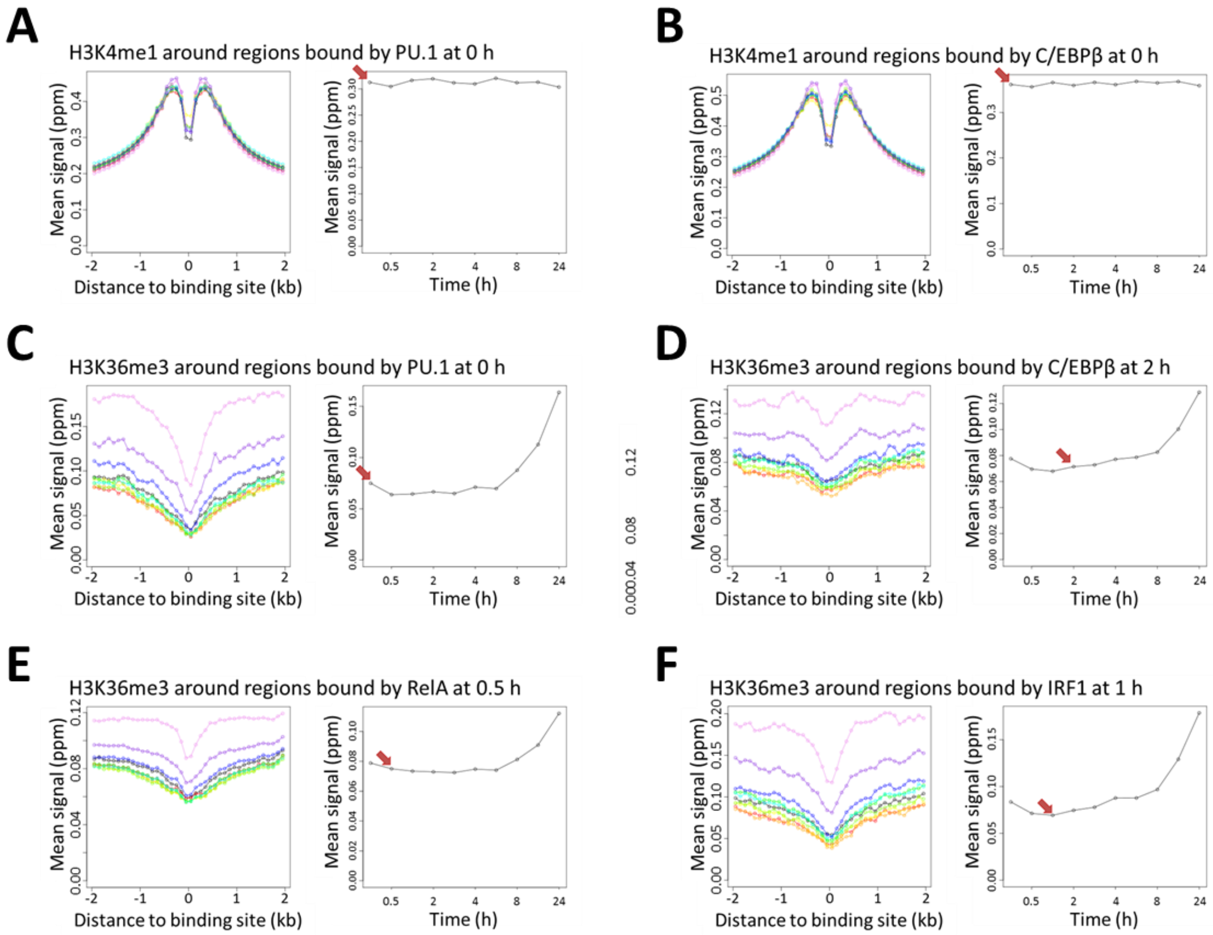

Fig. S15: Interaction between TF binding and histone modifications. (A) For all genomic regions that are pre-bound by PU.1 (0h), mean H3K4me1 signals are shown over time. Left: profile of mean values (y axis) over time in bins of 100 bps in function of distance (x axis) to the TF binding site. Line colors are as in Fig. 4A. Right: mean values (y axis) summed over the region -2kb to +2kb over all bound regions, over time (x axis). The red arrow indicates the time at which these regions become bound by PU.1. (B) Same as (A) for C/EBPβ and H3K4me1. (C-F) Same as (A) for H3K36me3 around regions bound by PU.1, C/EBPβ, RelA, and IRF1.

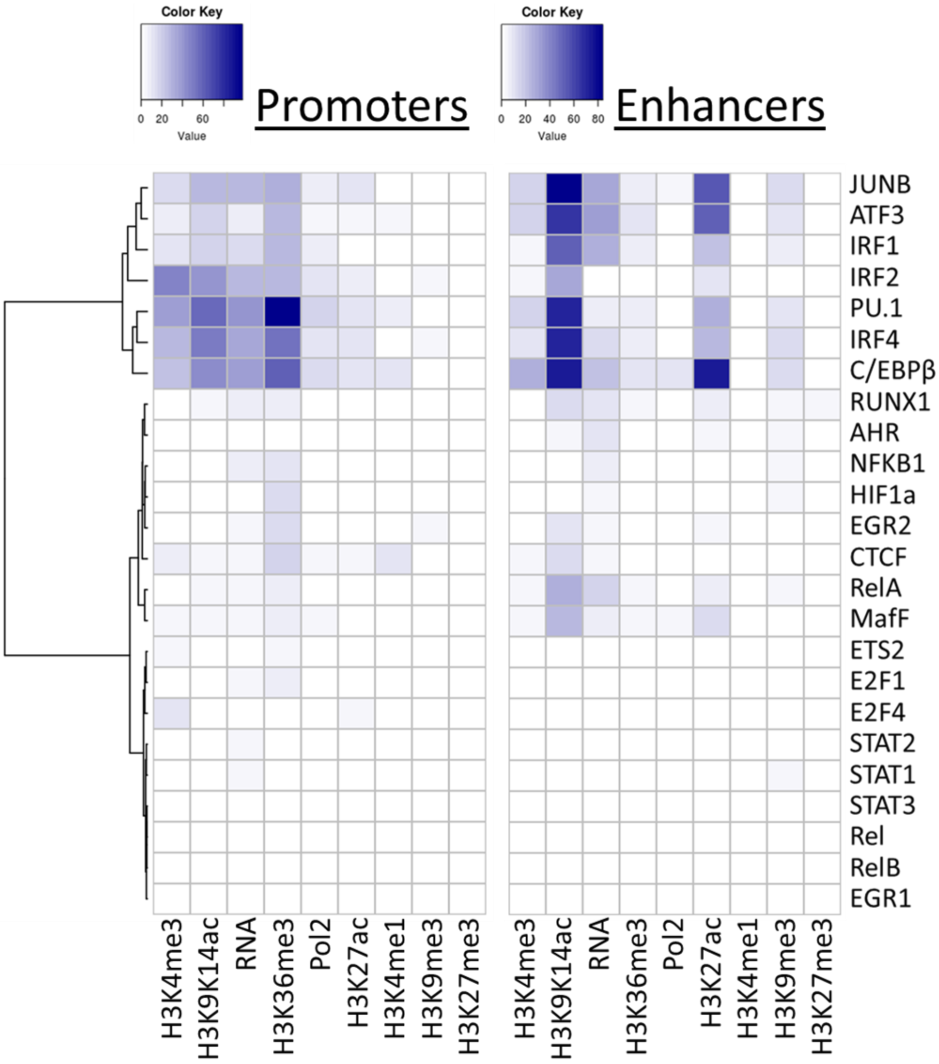

Fig. S16: Associations between pre-stimulation TF binding at promoters (left) and enhancers (right), and LPS-induced increases in histone modifications, Pol2 binding and transcription at the newly bound regions. Colors in the heatmap represent the degree of co-incidence (Fisher's exact test, -$\log_{10}$ p values) between pre-stimulus TF binding (rows) and post-stimulus increases (columns). TFs (rows) have been grouped through hierarchical clustering by similarity of their association pattern.

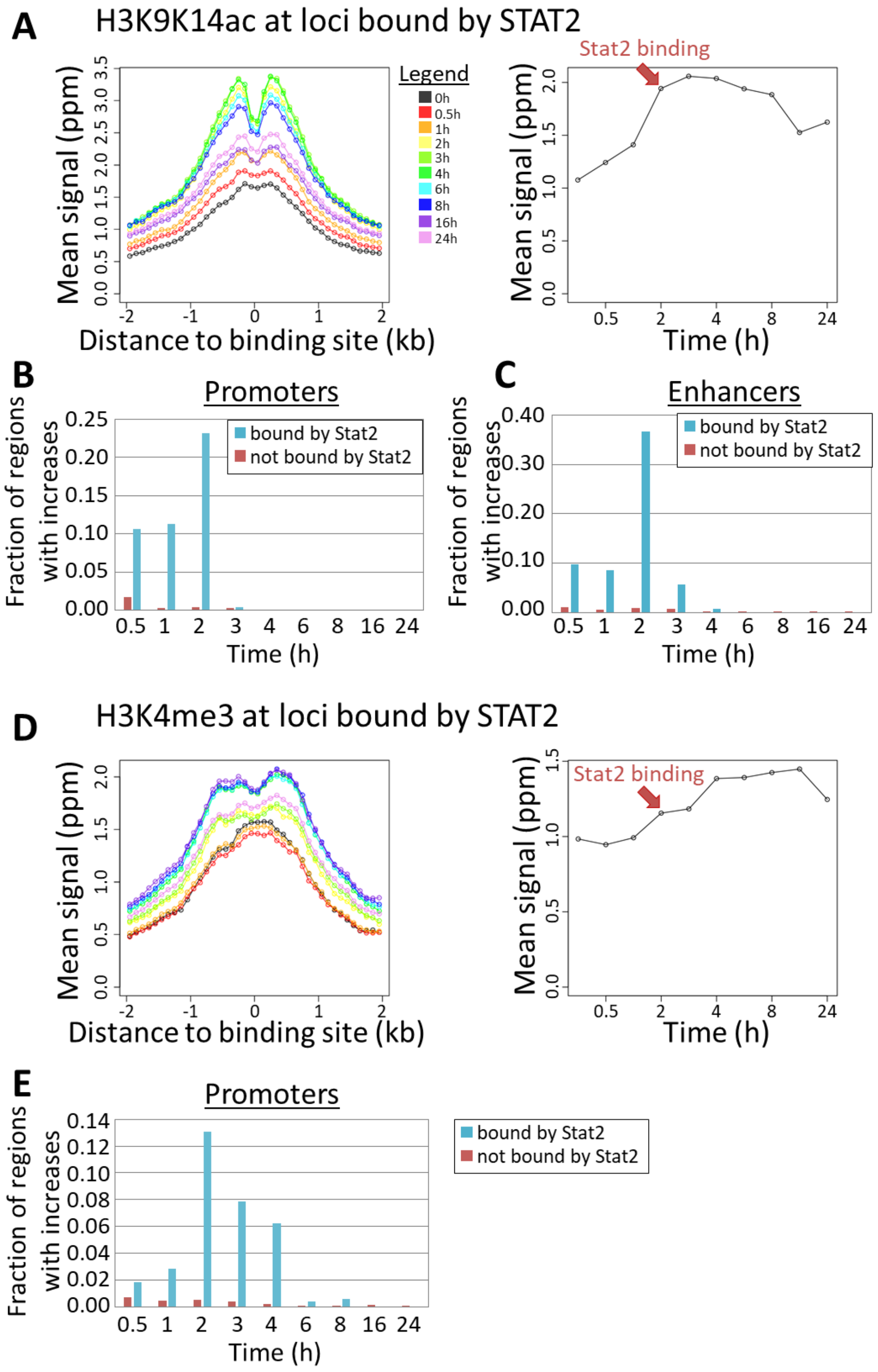

Fig. S17: Interaction between STAT2 binding and histone modifications over time. The plots shown are the same as shown in Fig. 4 for STAT1 bound regions. The numbers of promoters shown in (B) and (E) are 497 promoters bound by STAT2 at 2h, and 23,879 promoters not bound by STAT2. In (C) there are 267 enhancers bound by STAT2 at 2h, and 33,801 not bound by STAT2.

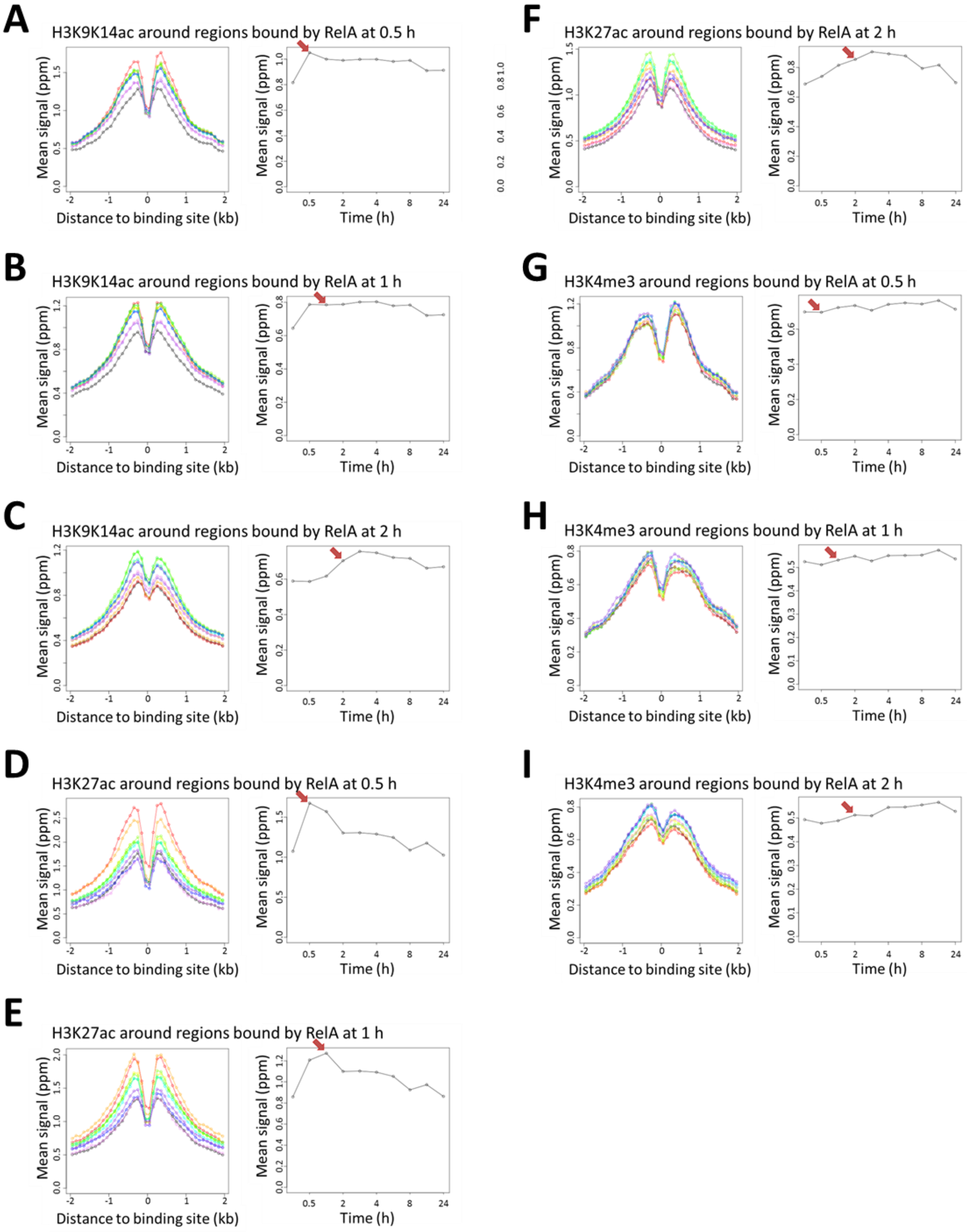

Fig. S18: Interaction between RelA binding and histone modifications. (A-C) For all genomic regions bound by RelA at 0.5h (A), 1h (B), and 2h (C) after LPS stimulation, mean H3K9K14ac signals are shown over time. Left: profile of mean values (y axis) over time in bins of 100 bps in function of distance (x axis) to the TF binding site. Line colors are as in Fig. 4A. Right: mean values (y axis) summed over the region -2kb to +2kb over all bound regions, over time (x axis). The red arrow indicates the time at which these regions become bound by RelA. (D-F) Similar as (A-C) for H3K27ac. (G-I) Similar as (A-C) for H3K4me3.

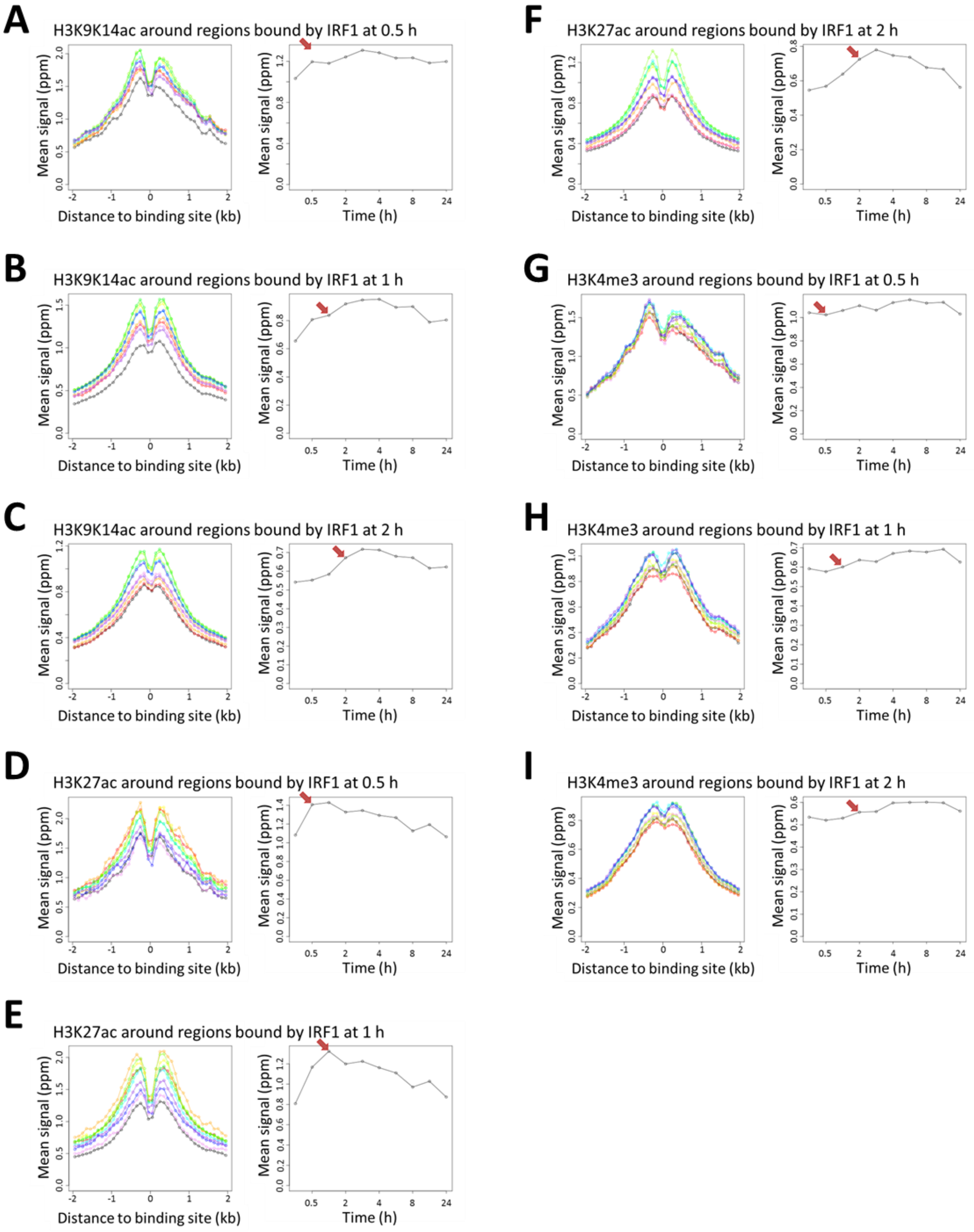

Fig. S19: Interaction between IRF1 binding and histone modifications. (A-C) For all genomic regions bound by IRF1 at 0.5h (A), 1h (B), and 2h (C) after LPS stimulation, mean H3K9K14ac signals are shown over time. Left: profile of mean values (y axis) over time in bins of 100 bps in function of distance (x axis) to the TF binding site. Line colors are as in Fig. 4A. Right: mean values (y axis) summed over the region -2kb to +2kb over all bound regions, over time (x axis). The red arrow indicates the time at which these regions become bound by IRF1. (D-F) Similar as (A-C) for H3K27ac. (G-I) Similar as (A-C) for H3K4me3.

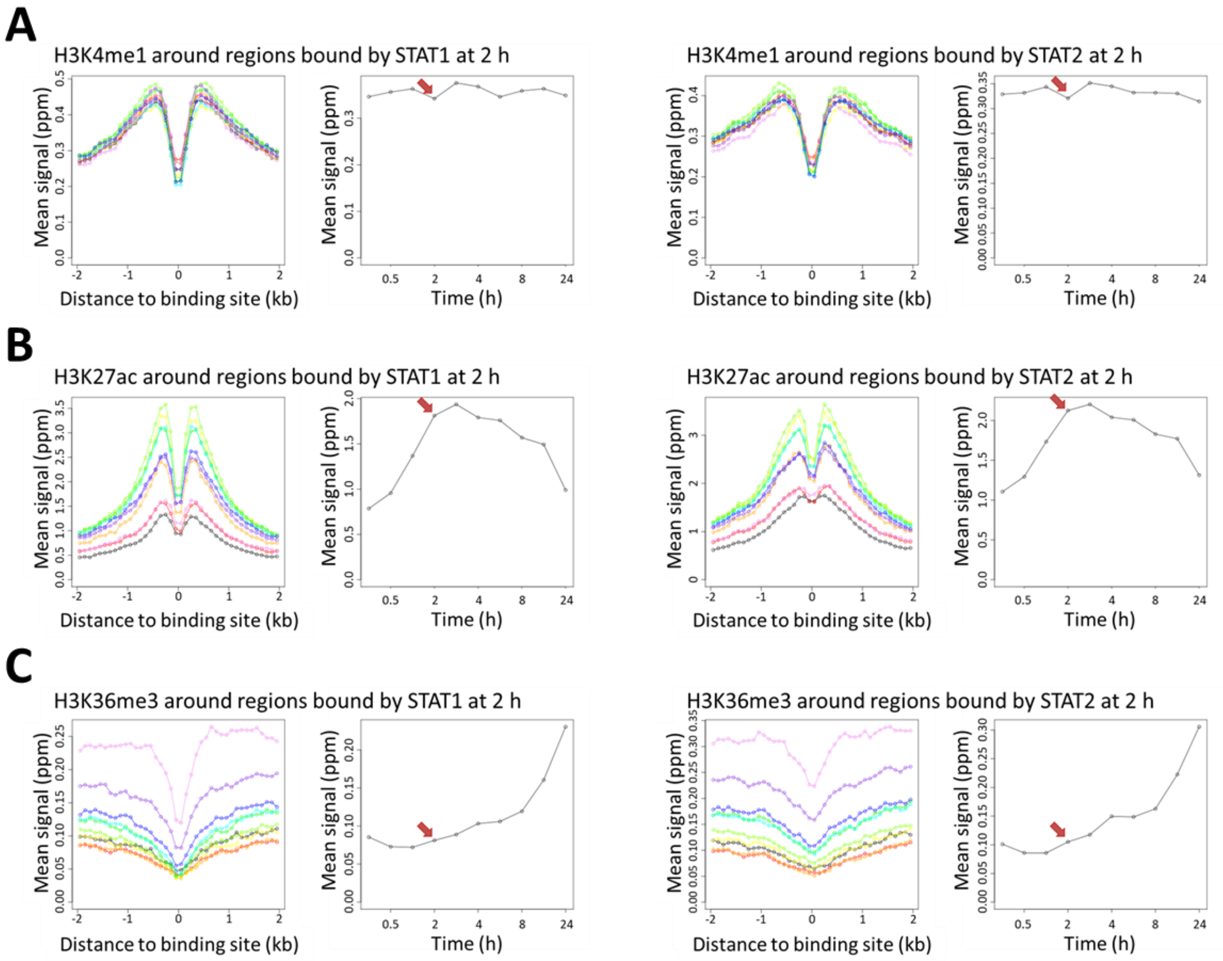

Fig. S20: Interaction between STAT1/STAT2 binding and histone modifications over time. The plots shown are similar to those shown in Fig. 4 for STAT1-bound regions, and in Fig. S14 for Stat2-bound regions. Plots are shown for H3K4me1 (A), H3K27ac (B), and H3K36me3 (C), for STAT1-bound (left) and STAT2-bound (right) regions.

Fig. S21: (next page) Increase of H3K9K14ac and H3K4me3 at STAT1/2-bound promoters of TRIF-dependent genes. (A) Heatmap showing gene expression changes in WT, *Myd88*$^{-/-}$, and *Trif*$^{-/-}$ mice after LPS stimulation of mouse DCs for TRIF-dependent genes. (B) TF binding ratios of promoter regions with stable expression (“unchanged”, blue), all LPS-induced promoters in WT (red), TRIF-dependent promoters (green), and MyD88-dependent promoters (purple). TRIF-dependent promoters are often bound by STAT1 and/or STAT2. (C) Fraction of regions with increases in H3K9K14 for TRIF- and MyD88-dependent promoters. For TRIF-dependent genes, plots are also shown specifically for STAT1 and/or STAT2 bound and unbound regions. Colors of bars indicate the timing of H3K9K14ac induction. (D) Same as (C) for H3K4me3 induction. Increases in H3K9K14ac and H3K4me3 are highly specific for STAT1/2 bound TRIF-dependent genes, and concentrated at time points 0.5-2 h and 2-4 h, respectively. Percentages indicate the fraction of regions with induction times between 0.5-2 hours for H3K9K14ac, and between 2-4 hours for H3K4me3.

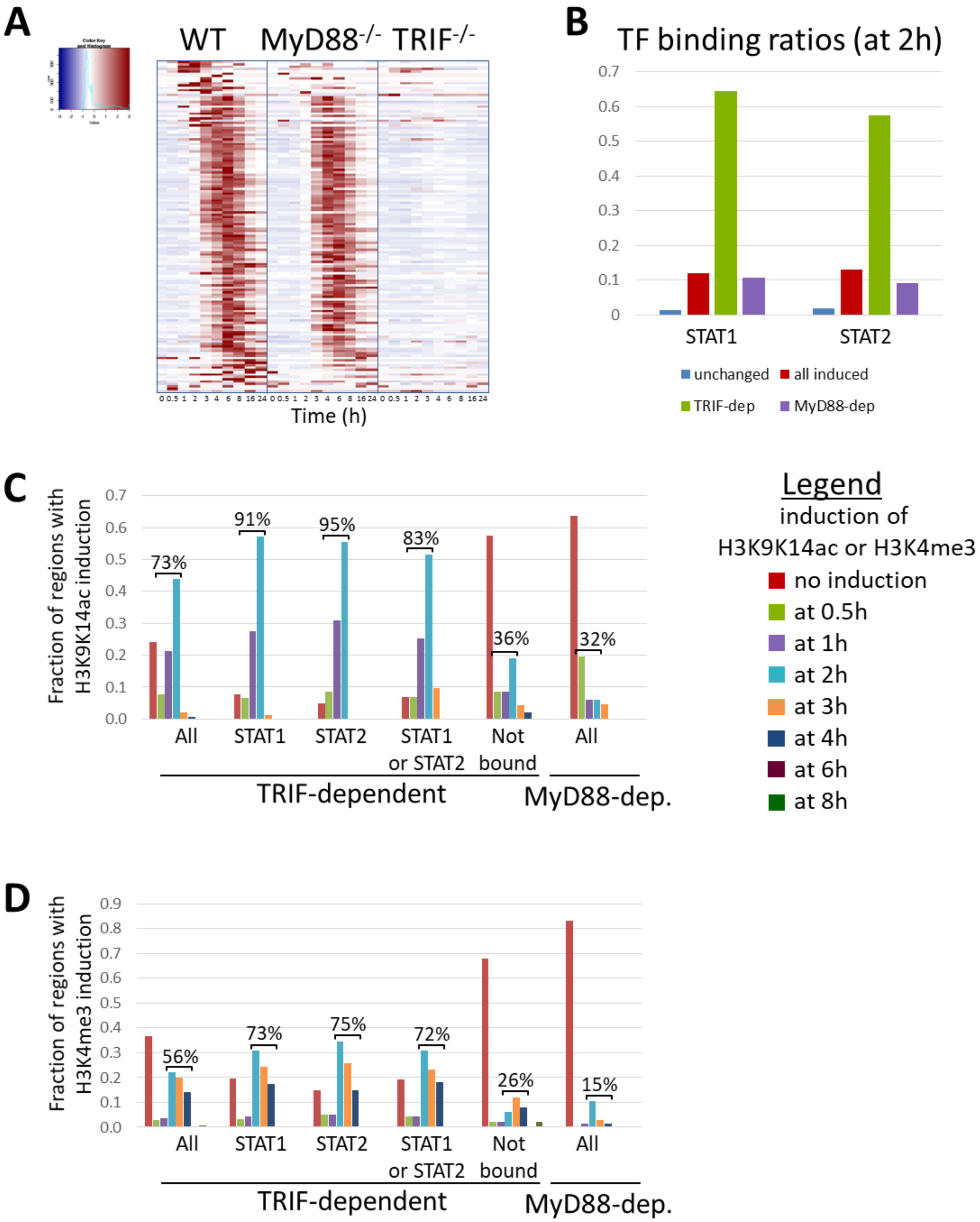
A
WT
MyD88-/-
TRIF-/-
Time (h)
B
TF binding ratios (at 2h)
STAT1
STAT2
unchanged
all induced
TRIF-dep
MyD88-dep
C
Fraction of regions with H3K9K14ac induction
73%
91%
95%
83%
36%
32%
All
STAT1
STAT2
STAT1 or STAT2
Not bound
All
TRIF-dependent
MyD88-dep.
Legend
induction of H3K9K14ac or H3K4me3
no induction
at 0.5h
at 1h
at 2h
at 3h
at 4h
at 6h
at 8h
D
Fraction of regions with H3K4me3 induction
56%
73%
75%
72%
26%
15%
All
STAT1
STAT2
STAT1 or STAT2
Not bound
All
TRIF-dependent
MyD88-dep.

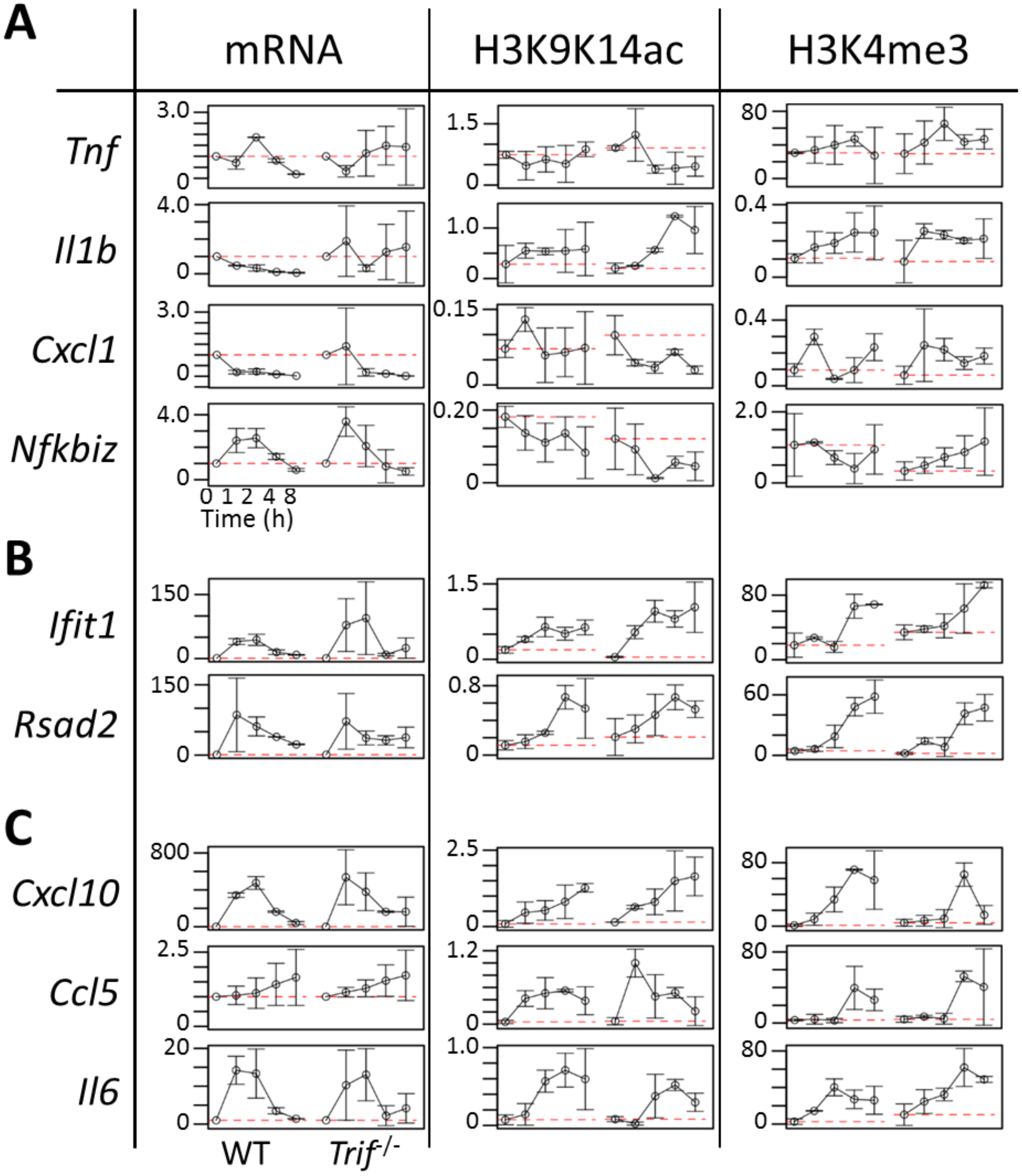

Fig. S22: Gene expression (mRNA), H3K9K14ac and H3K4me3 dynamics in WT, and *Trif*$^{-/-}$ cells following IFN-β stimulation. Genes are divided into three groups as in Fig. S11. Induction of expression and accumulation of H3K9K14ac and H3K36me3 is observed predominantly in genes under (B) and (C), but not those under (A). Accumulation of histone modifications is not affected in *Trif*$^{-/-}$ cells. Error bars represent the standard deviation based on duplicate experiments. The red dotted line in each graph represents the mean value at 0h. Y axes represent fold induction (for mRNA) and % input (for H3K9K14ac and H3K4me3).

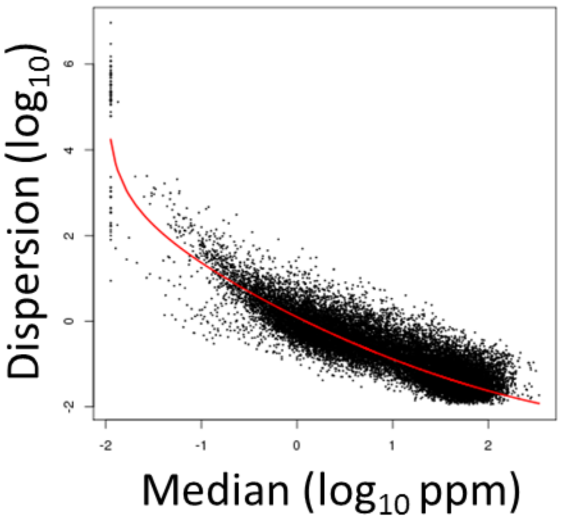

Fig. S23: Plot of median versus dispersion for H3K9K14ac reads (ppm) at the genome-wide set of promoters and enhancers. Each dot represents the median and dispersion of H3K9K14ac signals over the 10 time points, for 1 region. The red line is a plotted second order polynomial.
